# Supplementary material for: High-Throughput Screening of FDA-Approved Drug Library Reveals Ixazomib Is a Broad-Spectrum Antiviral Agent against Arboviruses
Source: Viruses. 2022 Jun 24;14(7):1381. doi: 10.3390/v14071381 (PMC9322861; doi:10.3390/v14071381)
Supplement: Supplementary file 1 [file viruses-14-01381-s001.zip › viruses-1745077-supplementary.pdf]

Table S1. Compounds against TBEV, WNV, YFV and CHIKV.

Pathway	Molecular Name	Chemical Structure	Target
**RTK (FLT3 EGFR Akt MEK) inhibitors**			
	Cabozantinib malate (XL184)	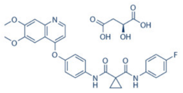	VEGFR2,c-Met, Ret, Kit, Flt-1/3/4, Tie2, AXL
	Olmutinib (BI 1482694)	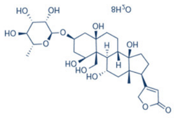	EGFR
	Pazopanib HCl (GW786034 HCl)	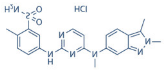	VEGFR1/2/3, PDGFR, FGFR, c-Kit, c-fms
	Cabozantinib (BMS-907351)	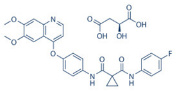	VEGFR2, c-Met, Ret, Kit, Flt-1/3/4, Tie2, AXL
	Regorafenib (BAY 73-4506)	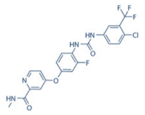	VEGFR1/2/3, PDGFR-β, Kit (c-Kit), RET (c-RET), Raf-1
	Sorafenib (BAY 43-9006) tosylate	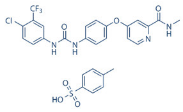	Raf-1, B-Raf, VEGFR2, VEGFR3, PDGFR-β, Flt-3, c-KIT
	Nintedanib (BIBF 1120)	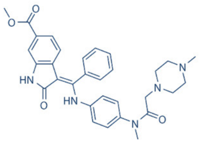	VEGFR1/2/3, FGFR1/2/3, PDGFRα/β
	Tivozanib (AV-951)	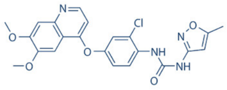	VEGFR1/2/3, PDGFR, c-Kit, FGFR-1, Flt3, c-Met, EGFR, IGF-1R
	Regorafenib (BAY-734506) Monohydrate	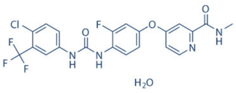	VEGFR1/2/3, PDGFR-β, c-Kit, c-RET, RAF-1, B-RAF, B-RAF
	Osimertinib mesylate	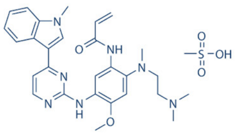	EGFR
	Sorafenib (BAY 43-9006)	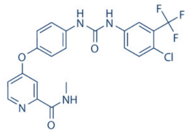	Raf-1, B-Raf, VEGFR-2, VEGFR-3, PDGFR-β, Flt-3, c-KIT

Crizotinib (PF-02341066)	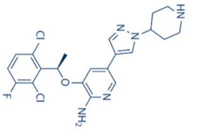	c-Met, ALK
Venetoclax (ABT-199)	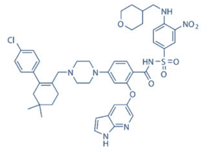	Bcl-2
Bosutinib (SKI-606)	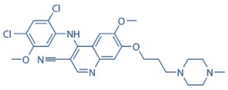	Src/Abl
**20S proteasome β5 inhibitors**		
Carfilzomib (PR-171)	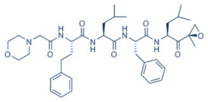	20S proteasome β5
Ixazomib (MLN2238)	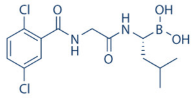	20S proteasome β5
Ixazomib Citrate	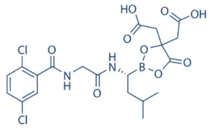	20S proteasome β5
**DNA synthesis inhibitors**		
Doxorubicin (Adriamycin) HCl	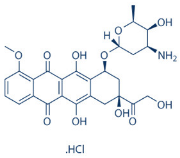	DNA topoisomerase II
Gemcitabine HCl	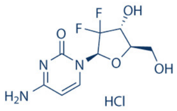	DNA synthesis
Gemcitabine	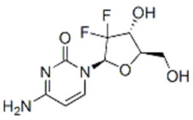	DNA synthesis
Proflavine Hemisulfate	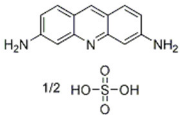	DNA synthesis
Pirarubicin	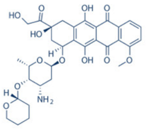	DNA topoisomerase II

Epirubicin (IMI 28) HCl	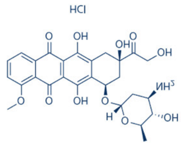	Topoisomerase
**Ion channel inhibitors**		
Rotenone (Barbasco)	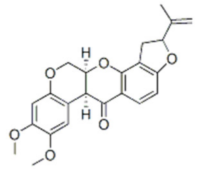	NADH/DB oxidoreductase, NADH oxidase
Hexachlorophene	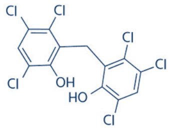	KCNQ1/KCNE1
Ouabain	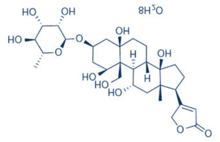	Na+/K+-ATPase
Azelnidipine	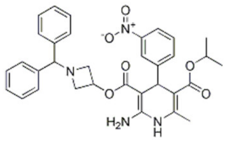	Calcium channel
Digoxin (NSC 95100)	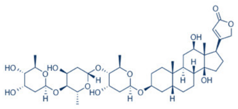	Na+/K+-ATPase
**Antiparasitics, antibacteria drugs**		
Nitazoxanide	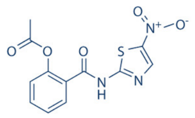	mTORC1
Temsirolimus (CCI-779)	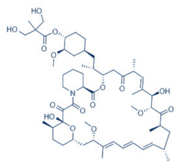	mTOR
Salinomycin	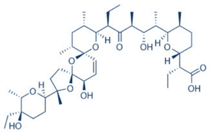	
Buparvaquone	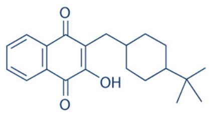	

pyrvinium	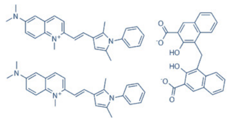	
Isavuconazole	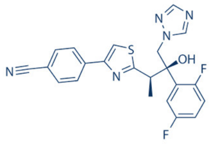	14α-lanosterol
Quinacrine 2HCl	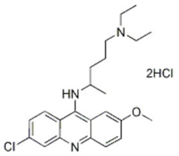	phospholipase A2
Atovaquone	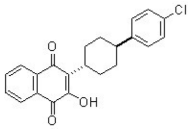	
Mebendazole	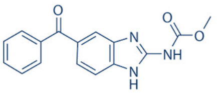	
Cloxiquine	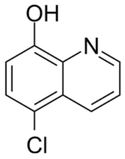	
Bithionol	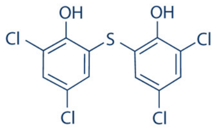	sAC
Ethacridine lactate monohydrate	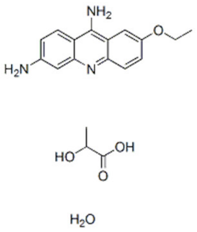	
**Natural products**		
Homoharringtonine	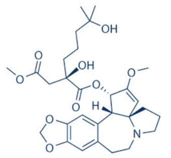	ribosomal A-sit
Harringtonine	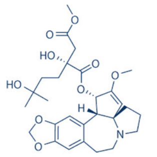	

Saikosaponin D	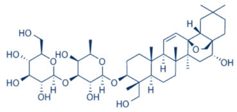	
Lanatoside C	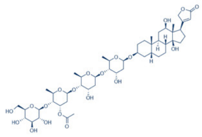	
Halofuginone	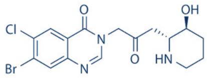	prolyl-tRNA synthetase
**Others**		
Mycophenolate Mofetil	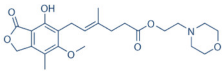	inosine monophosphate dehydrogenase I/II
CB-5083	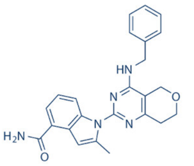	p97 AAA ATPase
Verteporfin	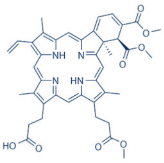	YAP-TEAD
Mycophenolic acid	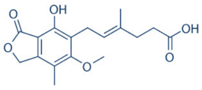	IMPDH
Posaconazole (SCH 56592)	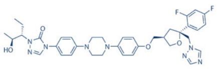	CYP3A4
Ivacaftor (VX-770)	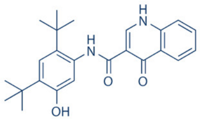	CFTR
Pimozide	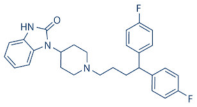	Dopamine Receptors

Telotristat Etiprate	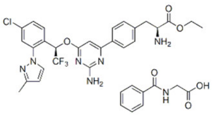	tryptophan hydroxylase
Sonidegib (NVP-LDE225)	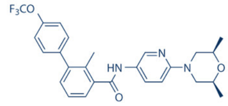	Smoothened
Methylene Blue	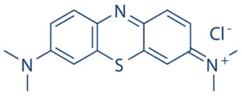	tau,sAC
Colchicine (NSC 757)	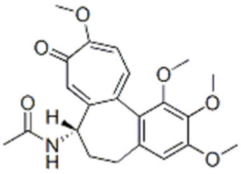	
Simeprevir (TMC435)	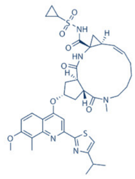	hepatitis C virus (HCV) NS3/4A protease
Sofosbuvir (GS-7977)	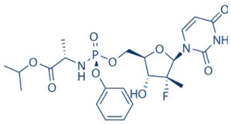	HCV NS5B polymerase
Desogestrel	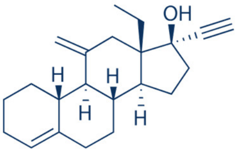	
Cetylpyridinium Chloride	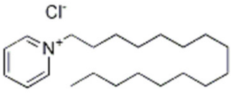	
